# 阿帕替尼对肺癌细胞侵袭迁移的影响及其作用机制

**DOI:** 10.3779/j.issn.1009-3419.2019.05.02

**Published:** 2019-05-20

**Authors:** 茵 袁, 颢 宫, 永文 李, 洪兵 张, 颖 李, 伟婷 李, 攀 王, 睿峰 施, 红雨 刘, 军 陈

**Affiliations:** 1 300052 天津，天津医科大学总医院肺部肿瘤外科 Department of Lung Cancer Surgery, Tianjin 300052, China; 2 天津市肺癌研究所，天津市肺癌转移与肿瘤微环境重点实验室 Tianjin Key Laboratory of lung Cancer Metastasis and Tumor Microenvironment, Tianjin Lung Cancer Institute, Tianjin Medical University General Hospital, Tianjin 300052, China

**Keywords:** 阿帕替尼, 肺肿瘤, 侵袭, 迁移, Apatinib, Lung neoplasms, Invasion, Migration

## Abstract

**背景与目的:**

肺癌是世界上对人类健康产生重大危害的癌症之一，近年来靶向治疗的效果日益显著。阿帕替尼（Apatinib, YN968D1）是目前研究较热的多靶点抗肿瘤药物，本研究旨在探讨Apatinib对肺癌细胞生物学特性的影响和其可能的作用机制。

**方法:**

体外培养肺腺癌细胞株H1299与H3255，CCK法、划痕实验及Transwell实验检测Apatinib对H1299与H3255细胞增殖、迁移及侵袭的影响，Western blot检测肿瘤血管生成和侵袭相关蛋白的表达。

**结果:**

Apatinib显著抑制H1299与H3255的增殖、迁移及侵袭能力，并呈浓度依赖性。Western blot显示，随药物浓度增加，VEGF、VEGFR2、N-cadherin、MMP9、MMP2、Vimentin表达下调，E-cadherin表达上调。

**结论:**

Apatinib可抑制肺腺癌细胞H1299、H3255的侵袭迁移能力，其机制可能通过调控上皮-间充质转化及基质金属蛋白酶相关蛋白的表达来实现。

## Abstract (en)

**Background and objective:**

Lung cancer is one of the most deadly cancers in the world for human. In recent years, the effect of targeted therapy has become increasingly significant. Apatinib is a multi-target anti-tumor drug that is currently under study. The purpose of this study is to investigate the effects of Apatinib on the biological characteristics of lung cancer cells and its possible mechanism.

**Methods:**

Lung cancer cell lines H1299 and H3255 were cultured *in vitro*. The effects of Apatinib on proliferation, migration and invasion of H1299 and H3255 cells were detected by cell proliferation assays wound healing assays and Transwell assays. The protein expression related to cancer angiogenesis and invasion was detected by Western blot.

**Results:**

Apatinib significantly inhibited the proliferation, migration and invasion of H1299 and H3255 in a concentration-dependent manner. Western blot showed that with the increasing of drug concentration, VEGF, VEGFR2, N-cadherin, MMP9, MMP2 and Vimentin were down-regulated, and E-cadherin were up-regulated.

**Conclusion:**

Apatinib can inhibit the invasion and migration of lung adenocarcinoma cells H1299 and H3255. By regulation of epithelial-mesenchymal transition and the expression of matrix metalloproteinase-related proteins.

肺癌是目前世界上最重要和致命的癌症之一，其死亡病例占所有癌症死亡人数的25%。在所有肺癌病例中，非小细胞肺癌（non-small cell lung cancer, NSCLC）占80%-85%。NSCLC包括腺癌、鳞癌和大细胞肺癌三种主要的组织类型，其中腺癌约占40%-50%，鳞癌约占20%-30%^[1-3]^。只有少部分NSCLC患者在早期（Ⅰ期或Ⅱ期）诊断；超过60%的肺癌患者在诊断时肿瘤已出现侵袭或转移性病变（Ⅲ期或Ⅳ期）^[2]^。在过去的20年里，尽管肺癌的诊断和治疗技术得到了较大的进步，但肺癌的5年生存率仍然在10%-17%之间。其原因在于，多数的患者发生了转移。约30%的患者在初诊时就有远处转移，50%-60%的患者在治疗过程中发生转移，80%-90%的患者最终死于转移，因此，转移是肺癌患者最严重的挑战，是肺癌患者死亡的最重要原因^[4]^。

肿瘤的血管生成（angiogenesis）是肿瘤发展中的一个重要过程。肿瘤的生长有两个明显不同的阶段，即从无血管的缓慢生长阶段转变为有血管的快速增殖阶段，血管生成使肿瘤能够获得足够的营养物质，并且促进肿瘤的侵袭转移，是肿瘤生长和发展的关键环节。其中，血管内皮生长因子（vascular endothelial growth factor, VEGF）可刺激内皮细胞增殖与分化，是胚胎发生、骨骼生长期间生理性血管生成的关键调节因子。在近年研究中，发现VEGF和血管内皮生长因子受体（vascular endothelial growth factor receptor, VEGFR）在肿瘤发生发展过程中是促进肿瘤血管生成的重要因子，VEGF可通过VEGFR2正反馈作用刺激VEGF产生，从而促进肿瘤血管生成，进而促进肿瘤的活性及转移，因此，针对VEGF/VEGFR的治疗成为重要的肿瘤治疗方法之一^[5]^。

阿帕替尼（Apatinib, YN968D1）是一种新型多靶点抗肿瘤药物，其作为VEGFR2酪氨酸激酶抑制剂，在体内及体外均有很高的活性, 可通过抑制VEGF/VEGFR2信号通路抑制血管生成^[6]^。最近研究^[7-12]^表明，Apatinib对VEGFR2酪氨酸激酶抑制性治疗在胃癌、骨肉瘤、胆管癌、肝癌、结肠癌等多种肿瘤中有效，其机制不仅抑制血管生成，还可能与抑制肿瘤细胞侵袭迁移作用有关，其可能通过的途径为：通过STAT3抑制上皮-间充质转化（epithelial-mesenchymal transition, EMT）；抑制VEGFR2/RAF/MEK/ERK及PI3K/AKT/mTOR通路；与KIF5B-RET在肿瘤中的驱动因素相关等。并且，Apatinib可与表皮生长因子受体（epidermal growth factor receptor, EGFR）酪氨酸激酶抑制剂吉非替尼（Geftininb）联用，在*EGFR*基因T790M突变的吉非替尼耐药NSCLC细胞中，明显增强抗肿瘤作用^[13]^。上述研究表明，Apatinib不但可以抑制血管生成进而抑制肿瘤的发生发展，还可通过多种途径抑制肿瘤的转移。目前，我们对该药物在NSCLC中作用机制的了解仍然有限。本研究通过体外实验探讨Apatinib对肺腺癌细胞侵袭迁移能力的影响，并进一步探索其潜在的下游调控机制。

## 1 材料与方法

### 1.1 细胞系及主要试剂

人肺腺癌细胞系H1299（EGFR wt, N-Ras Q61K）与H3255（EGFR L858R, K-Ras wt）购自中国科学院细胞库（中国上海），阿帕替尼(S7297，Apatinib)购自Selleck公司，抗体: VEGF、VEGFR 2抗体购自Abcam公司，MMP9、MMP2抗体购自Santa Cruz公司，E-cadherin、N-cadherin、Vimentin、GAPDH抗体购自Cell Signaling Technology公司。RPMI-1640培养基、胎牛血清购自GIBCO公司。胰蛋白酶、CCK-8均购自Bioind公司。Tranwell购自美国Corning公司。二甲基亚砜（Dimethyl sulfoxide, DMSO）购自碧云天公司。

### 1.2 细胞培养

细胞培养于含10%胎牛血清的RPMI-1640培养基中，置于37 ℃、5%CO_2_饱和湿度的培养箱中，待细胞融合度达至90%左右，用0.25%胰酶-EDTA（ethylenedia-minetetracetic acid）消化传代，所有实验均采用对数生长期细胞。

### 1.3 CCK 8法测定细胞活性

收集H1299与H3255呈对数期稳定生长的细胞，调整细胞悬液浓度至2.5×10^4^/mL，向96孔板中每孔加入200 μL悬液，5%CO_2_，37 ℃培养过夜，待细胞贴壁后，各组加入浓度为0 μmol/L、2 μmol/L、4 μmol/L、8 μmol/L、16 μmol/L、32 μmol/L、64 μmol/L、128 μmol/L梯度递增的Apatinib 200 μL，每个浓度设4个复孔，5%CO_2_ 37 ℃孵育24 h后，吸去孔内培养基，每孔加入10 μL CCK-8及90 μL完全培养基，染色1 h；多功能酶标仪以OD 450nm处测量各孔吸光值；计算细胞抑制率及IC_50_。

### 1.4 划痕实验

将H1299与H3255细胞接种至6孔板中，加入完全培养基，待细胞汇合率为85%-95%时，用200 μL黄色枪尖划痕，吸去孔内培养基，PBS洗3次，将各孔分为对照组（NC）与不同浓度加药组，H1299细胞各组每孔分别加入0 μmol/L、8 μmol/L、16 μmol/L Apatinib 2mL，H3255细胞中各组每孔分别加入0 μmol/L、4 μmol/L、8 μmol/L Apatinib 2 mL于0 h、12 h、24 h时置于倒置显微镜下观察拍照。细胞迁移率=100%×（0 h划痕宽度-12 h或24 h划痕宽度）/0 h划痕宽度。每个实验独立重复3次，取平均值。

### 1.5 侵袭实验

准备Transwell及24孔板。按照1:10比例稀释基质胶并加入20 μL至Transwell小室上室，置于37 ℃温箱凝固4 h。收集加入完全培养基与加入Apatinib 8 μmol/L培养24 h的H1299与H3255细胞，分为对照组（NC）与加药组，使用无血清RPMI-1640培养基调整细胞浓度为1×10^5^/mL，取200 μL加入上室，将500 μL含10%胎牛血清的RPMI-1640培养基加入下室，培养箱培养24 h后取出小室，棉签轻柔擦去上室面细胞，4%多聚甲醛固定，结晶紫染色，PBS洗涤3遍。将小室置于倒置显微镜下观察，每个样本随机选取5个视野计数。每个实验独立重复3次，取平均值。

### 1.6 蛋白质免疫印记实验

将H1299与H3255细胞接种至6孔板中，待细胞汇合率为70%-80%时加入不同浓度Apatinib，24 h后提取蛋白。BCA法测蛋白浓度。加入SDS-PAGE蛋白上样缓冲液，充分混匀，100 ℃金属浴变性15 min。每种样品取30 μg蛋白上样电泳，采用湿转法电转移至PVDF膜，含5%脱脂牛奶的TBST摇床上室温封闭2 h。将PVDF膜按照不同分子量切开，加入对应的抗体，4 ℃摇床孵育过夜。次日用TBST室温漂洗3次，每次5 min，加HRP标记的二抗，室温孵育1 h，显色曝光，分析条带吸光值。

### 1.7 统计学分析

应用SPSS 19.0版软件进行统计分析，GraphPad Prism 5.01版软件处理数据分析作图，数据采用均数±标准差表示，实验中组间比较采用两样本均数*t*检验，以*P* < 0.05为差异有统计学意义。

## 2 结果

### 2.1 Apatinib能抑制肺癌细胞H1299与H3255的活性

如图 1所示，在H1299与H3255细胞中，经过不同浓度的Apatinib处理24 h后，Apatinib的IC_50_分别为（32.67±3.12）μmol/L和（14.44±1.92）μmol/L，而且两个细胞的活性随着Apatinib药物浓度的升高而呈明显下降趋势，提示Apatinib能够明显抑制肺腺癌细胞H1299与H3255的活性。

**1:**
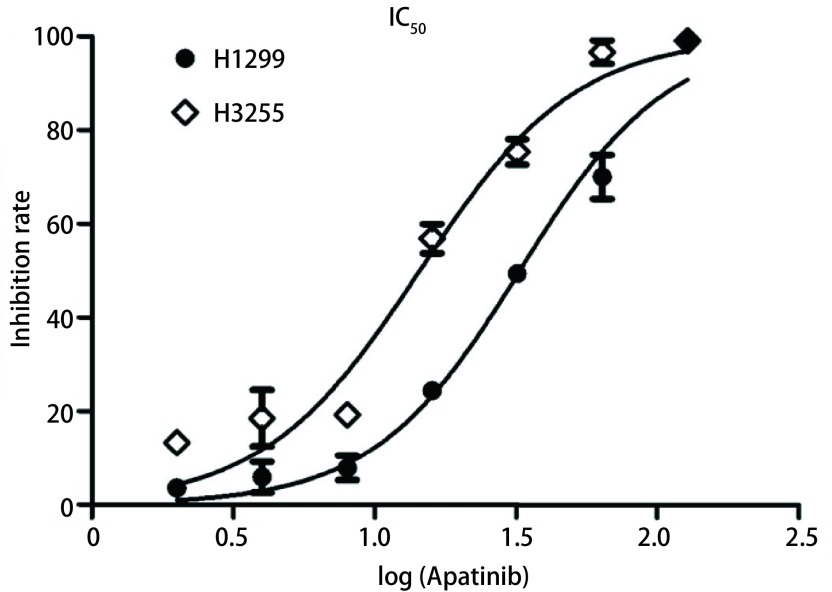
Apatinib抑制肺癌细胞增殖。不同浓度的Apatinib处理肺癌细胞H1299与H3255 24 h后，肺癌细胞的活性随药物浓度梯度的增加而明显降低。其中，H1299的IC_50_为（32.67±3.12）*μ*mol/L，H3255的IC_50_为（14.44±1.92）*μ*mol/L。 Apatinib inhibits lung cancer cell proliferation. After treatment with different concentrations of Apatinib for 24 h in lung cancer cells H1299 and H3255, the viability of lung cancer cells were decreased significantly with the increase of drug concentration gradient. The IC_50_ of H1299 is (32.67±3.12) *μ*mol/L, and the IC_50_ of H3255 is (14.44±1.92) *μ*mol/L.

### 2.2 Apatinib能抑制肺癌细胞H1299与H3255的迁移和侵袭能力

为了研究Apatinib对肺癌细胞H1299与H3255的迁移和侵袭能力的作用，我们分别用不同浓度Apatinib处理H1299与H3255细胞，通过划痕实验分别在12 h和24 h节点检测Apatinib对细胞迁移能力的影响。如图 2A、图 2C所示，在H1299细胞中，与对照组（NC）相比，12 h时8 μmol/L Apatinib对细胞的迁移能力有一定的抑制作用，但无统计学意义；而16 μmol/L Apatinib对细胞的迁移能力则有明显的抑制作用（*P* < 0.05）；24 h时8 μmol/L与16 μmol/L Apatinib对细胞的迁移能力均有明显的抑制作用，且药物高浓度抑制作用比低浓度更加明显（*P* < 0.05）。如图 2B、图 2D所示，在H3255细胞中，与对照组（NC）相比，12 h与24 h时4 μmol/L与8 μmol/L Apatinib对细胞的迁移能力均有明显的抑制，而且药物高浓度的抑制作用比低浓度更明显（*P* < 0.05）。这些结果表明Apatinib具有抑制肺腺癌细胞迁移的能力。

**2:**
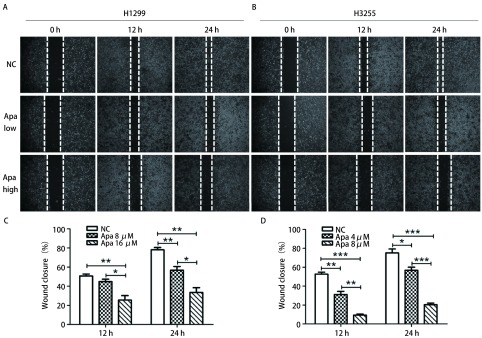
Apatinib抑制肺癌细胞迁移。A、C：H1299细胞划痕实验；B、D：H3255细胞划痕实验。经低浓度Apatinib（Apa low）与高浓度Apatinib（Apa high）处理24 h后，H1299与H3255细胞的划痕愈合度与对照组（NC）相比均显著降低，并且随着Apatinib的药物浓度增加，抑制作用增强。 Apatinib inhibits lung cancer cell migration. A, C: H1299 cell scratch test; B, D: H3255 cell scratch test. After treated with different concentrations of Apatinib (Apa) for 24 h, the scratch healing degree of H1299 and H3255 cells was significantly lower than that of the control group, and the inhibitory effect was enhanced with the increase of drug concentration of Apatinib.

进一步通过Transwell小室检测Apatinib对肺癌细胞H1299与H3255侵袭能力的影响。如图 3所示，H1299细胞中，每视野细胞数对照组（NC）为（220.7±18.49），Apatinib处理组为（101. 0±22.52）；H3255细胞中，每视野细胞数对照组（NC）为（149.3±14.72），Apatinib处理组为（67.00±23.96）。与对照组相比，H1299与H3255细胞中Apatinib处理组穿过基质胶的细胞均显著减少（*P* < 0.05）。这些结果表明Apatinib能够抑制肺腺癌细胞H1299与H3255的侵袭。

**3:**
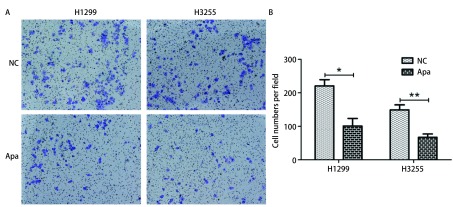
Apatinib抑制肺癌细胞侵袭能力。A, B：Transwell小室检测细胞侵袭能力，与对照组（NC）相比，Apatinib（Apa）处理后的穿过小室的H1299与H3255细胞数目明显减少。 Apatinib inhibits the invasive ability of lung cancer cells of H1299 and H3255. A-B: Compared with the control group, the number of cells that passed through the transwell after Apatinib (Apa) treatment was significantly reduced.

### 2.3 Apatinib抑制VEGF/VEGFR2和调控EMT及基质金属蛋白酶的表达

如图 4A-图 4C所示，经不同浓度Apatinib处理H1299与H3255细胞24 h后，通过Western blot检测蛋白表达情况发现，随药物浓度增加，VEGF、VEGFR2、N-cadherin、MMP9、MMP2、Vimentin表达下调，E-cadherin表达上调。提示Apatinib通过抑制VEGF/VEGFR2和调控EMT及基质金属蛋白酶等相关蛋白的表达，进而可能抑制细胞的迁移及侵袭。

**4:**
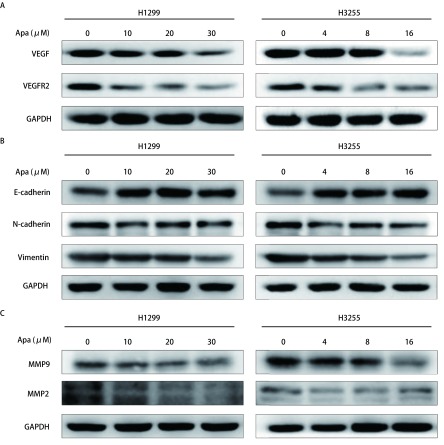
Apatinib处理不同肺癌细胞株后相关蛋白的变化情况。经不同浓度Apatinib处理H1299和H3255细胞24 h后，通过Western blot检测不同蛋白表达情况，其中，GAPDH为内对照。A：Apatinib抑制VEGF/VEGFR2蛋白的表达；B：Apatinib调节EMT蛋白的变化。随药物浓度增加，E-cadherin蛋白表达上调，而N-cadherin、Vimentin蛋白表达下调；C：Apatinib调节基质金属蛋白酶的变化。随药物浓度增加，MMP9、MMP2蛋白表达下调。 Alter the expression of the proteins after Apatinib treatment in different lung cancer cell lines. After treatment of H1299 and H3255 cells with different concentrations of Apatinib for 24 hours, the expression of different proteins was detected by Western blot, wherein GAPDH was an internal control. A: Apatinib inhibits the expression of VEGF/VEGFR2 protein; B: Apatinib regulates the expression of EMT proteins. The expression of E-cadherin protein was increased with the increasing of drug concentration of Apatinib, while the expression of N-cadherin and Vimentin protein was decreased; C: Apatinib regulates the expression of matrix metalloproteinases. The expression of MMP9 and MMP2 proteins was decreased with the increasing of drug concentration of Apatinib.

## 3 讨论

目前，全球每年约有180万人确诊为肺癌，160万人死于肺癌。肺癌患者的5年生存率从4%-17%不等，取决于临床分期和地域差异^[14]^。传统治疗方式为手术、放疗及化疗，近年新型的治疗方式以靶向治疗及免疫治疗为主，如针对*EGFR*突变采用Gefitinib、Osimertinib等靶向药物，提高了部分患者的生存时间，但长期使用靶向药物的同时肿瘤细胞耐药性随之产生，因此有待研发特异性靶向作用的下一代药物和更多特异性突变位点的靶向药物。相关药物如细胞周期抑制剂、血管生成抑制剂如PD0332991、Apatinib等与靶向药物的联用也越来越多的进行了探索^[2, 13, 15]^。而对于Apatinib的研究开始于胃癌方面，进而证实可抑制多种肿瘤的转移，但对肺癌细胞的相关作用报道尚少。所以，对于肺癌细胞及药物作用机制的研究仍然具有十分重要的意义。

VEGF通过自身与其受体功能对血管生成起重要作用，并且在体内及体外都是内皮细胞的存活因子，可通过PI3K-AKT途径减少细胞凋亡，同时诱导抗凋亡蛋白Bcl-2和A1的表达。还可以通过癌细胞本身存在的直接结合的VEGFR来调节肿瘤生长，包括多种实体肿瘤及血液系统恶性肿瘤。VEGF和VEGFR的过度表达与肿瘤生长速度、微血管密度、增殖、肿瘤转移潜能及各种恶性肿瘤患者预后不良有关。目前，针对VEGF及其受体的靶向药物研究正在进行。抗血管生成类药物如索拉非尼、苏尼替尼和帕唑帕尼在肾细胞癌、结肠癌等肿瘤治疗中取得成效。Apatinib是多靶点抗肿瘤药物，其主要功能为针对VEGFR酪氨酸激酶的产生抑制性，其结合亲和力是索拉非尼（Sorafenib）的10倍。Apatinib可在细胞中有效抑制VEGFR2、c-kit和c-src的激酶活性，并抑制VEGFR2、c-kit和PDGFRβ的磷酸化，进而可抑制人脐静脉内皮细胞的增殖及迁移。在体内实验中，Apatinib可以阻断大鼠主动脉环的形成，这是对其抗血管生成活性的更好验证^[5, 6]^。而临床中其靶向药物实验也在开展。Li等^[11]^将144例转移性胃癌且至少两种化学治疗方失败的患者纳入研究，患者被随机分配接受安慰剂（A组），Apatinib 850 mg每日一次（B组）或Apatinib 425 mg每日两次（C组）。结果显示Apatinib组与安慰剂组的中位生存时间显著延长，Apatinib已被证明是没有化疗适应症的晚期胃癌患者的唯一有效药物。随后，Apatinib在几项临床试验^[16-18]^中显示出对乳腺癌及NSCLC具有良好治疗效果。证明Apatinib可以对多种肿瘤产生抑制作用，延长患者生存期。

本实验结果显示Apatinib可抑制肺腺癌细胞H1299与H3255的增殖及侵袭迁移能力，并且其作用呈药物浓度依赖性，其作用机制可能与VEGF/VEGFR2、N-cadherin、MMP9、MMP2、Vimentin及E-cadherin有关。相关基础实验表明，Apatinib抑制VEGF-VEGFR2-PI3K-AKT信号传导，诱导人类肝内胆管癌细胞（intrahepatic cholangiocarcinoma, ICC）凋亡，并且最近数据^[19]^表明肿瘤细胞中的自分泌VEGF信号传导在促进其增殖和抑制细胞凋亡中起重要作用。而在胆管癌细胞中，Apatinib能抑制VEGFR2/RAF/MEK/ERK及PI3K/AKT/mTOR通路，降低转移相关蛋白如Slug、Snail和MMP9的表达，显著抑制VEGF介导的细胞迁移和侵袭^[8]^。并且，Apatinib可通过抑制VEGFR2/ERK通路促进食管癌、膀胱癌及肺癌细胞凋亡^[20-22]^。另外，Apatinib可在肺腺癌细胞A549中对KIF5B-RET驱动的肿瘤治疗中相关的迁移和侵袭产生抑制作用^[12]^。关于Apatinib的作用机制，Zheng等^[7]^在对骨肉瘤细胞的研究中发现，Apatinib在体外和体内通过STAT3抑制EMT和PD-L1，同时下调N-cadherin、MMP9、MMP2、Vimentin及上调E-cadherin的表达，进而明显抑制癌细胞的转移且无明显细胞毒性，对本实验研究方向给予提示。本研究表明，Apatinib抑制VEGF及VEGFR，并且通过诱导EMT相关信号蛋白:上调E-cadherin、下调N-cadherin维持细胞间紧密连接，下调Vimentin修复细胞骨架，调控上皮-间充质转化，从而抑制细胞侵袭迁移能力，并与金属蛋白酶MMP9、MMP2表达量下调相关。
